# Uganda chicken genetic resources: II. genetic diversity and population demographic history inferred from mitochondrial DNA D-loop sequences

**DOI:** 10.3389/fgene.2024.1325569

**Published:** 2024-03-07

**Authors:** Illyass Yussif, Donald Rugira Kugonza, Charles Masembe

**Affiliations:** ^1^ College of Agricultural and Environmental Sciences, Makerere University, Kampala, Uganda; ^2^ College of Natural Resources, Makerere University, Kampala, Uganda

**Keywords:** chicken mtDNA, D-loop, genetic differentiation, genetic relationship, phylogenetic structure, haplotype diversity, indigenous chickens

## Abstract

The genetic diversity of indigenous chickens, which comprise over 80% of the chicken resources in Uganda, is largely not well-characterized for their genetic contribution. This study assessed the genetic diversity and population structure of the indigenous chicken population in Uganda to serve as an essential component for improvement and conservation strategies. A set of 344 mitochondrial DNA (mtDNA) D-loop sequences among 12 Ugandan chicken populations was evaluated. Twenty-eight polymorphic sites, accounting for 4.26% of the total analyzed loci of 658 bp, defined 32 haplotypes. The haplotype diversity (*Hd*) was 0.437, with a nucleotide diversity (*π*) of 0.0169, while the average number of nucleotide differences (*k*) was 0.576, indicating a population that is moderately genetically diverse. Analysis of molecular variance found 98.39% (ρ < 0.01) of the total sequence variation among the chicken haplotypes within populations, 1.08% (ρ < 0.05) among populations, and 0.75% (ρ > 0.05) among populations within regions. This revealed subtle genetic differentiation among the populations, which appeared to be influenced by population fragmentation, probably due to neutral mutation, random genetic drift, and/or balancing selection. All the haplotypes showed affinity exclusively to the haplogroup-E mtDNA phylogeny, with haplotype UGA01 signaling an ancestral haplotype in Uganda. Neutrality tests Tajima’s D (−2.320) and Fu’s Fs (−51.369), augmented with mismatch distribution to measure signatures of recent historical demographic events, supported a population expansion across the chicken populations. The results show one matrilineal ancestry of Ugandan chickens from a lineage widespread throughout the world that began in the Indian subcontinent. The lack of phylogeographic signals is consistent with recent expansion events with extensive within-country genetic intermixing among haplotypes. Thus, the findings in this study hold the potential to guide conservation strategies and breeding programs in Uganda, given that higher genetic diversity comes from within the chicken population.

## Introduction

Diversity in domestic animals is an important component of global biodiversity and contributes to food security needs. It is generally understood that chickens in Uganda offer potential for meat and egg production (FAO, 2018) and are an important component of animal genetic resources (AnGRs) crucial to the economy of rural poor farmers in Uganda (FAO, 2009a). The chicken genetic resources in Uganda comprise over 48 million birds, which are largely composed of over 85% indigenous breeds distributed across all types of geographical regions of the country (Ssewannyana, 2004; MAAIF & UBOS, 2009). Specific indigenous chicken strains showing major phenotypic traits (normal feather distribution, frizzle-feather, naked-neck, tufted crest, feathered-shanks/ptilopody, and polydactyly chickens) are readily recognized (Ssewannyana et al., 2008; Semahoro et al., 2018). Several nondescript ecotypes based on the geographical location of the chicken (Ssewannyana, 2004) and anecdotal reports also exist. These offer genetic resources, which, when safeguarded, are key to future breeding programs in the face of environmental changes like climate change and emerging pests and diseases in chicken production. However, due to the slow growth rate, small body size, and low meat yield of the indigenous chicken breeds in Uganda, as compared to the exotic chickens, their commercial productions are generally sidelined and hardly able to compete with the exotic chickens (FAO, 2009b; Padhi, 2016). Consequently, traditional breeding methods, which only present a short-term solution for increasing productive performance, as well as continuous unstructured crossbreeding management, which has limited efficiency in genetically improving performance, may become rampant. Hence, there is an increased risk of losing indigenous chickens, which are resilient and well-adapted. It has been shown that centuries of exposure to diverse and stressful environmental conditions, including disease pathogens, heat stress, water scarcity, and poor quality feed, typical of the free-range/scavenging system, equip indigenous breeds with their unique adaptive traits (AU-IBAR, 2019; Hvilsom et al., 2022). In addition, continuous domestication and selective breeding ensured that animals with certain traits are kept for their genotype to become resourceful for improved adaptation to the low-input conditions prevailing in the free-range system (FAO, 2010). Therefore, to achieve economically and ecologically sustainable indigenous chicken production, genetic improvement of the relative performance of the indigenous chicken is emphasized. Improvement targeted at competitiveness under the socioeconomic circumstances of indigenous breed production environments is one of the practical options to ensure the conservation of genetic diversity and improvement for future sustainability (Koehler-Rollefson and Meyer, 2014). Ultimately, the prerequisite for the sustainable utilization and conservation of indigenous breeds is a thorough analysis of diversity up to the molecular level, which forms the basis for any population to evolve under natural adaptation, artificial selection, or both (FAO, 2011; Hvilsom et al., 2022).

Molecular markers have been used worldwide for the genetic assessment of the genetic structure of animal genetic resources. Information on the genetic structure, variation, and relationships of indigenous breeds at the molecular level, based on molecular tools, is necessary as a vital complement to the evaluation of phenotypic diversity and their interaction with the production systems (FAO, 2011; FAO, 2012). Nonetheless, the chicken diversity in Uganda remains poorly studied at the molecular level compared to other livestock species. An earlier study in Uganda using mitochondrial DNA (mtDNA) was limited in geographic scope, providing only an overview of the origin of East African chicken populations (Mwacharo et al., 2011). The enormous phylogenetic information contained in the mtDNA displacement (D)-loop makes it a good marker for intra- and interspecies genetic differentiation and phylogenetic relationship studies (Yamamoto, 2001). The use of an mtDNA marker has been recommended in the phylogeographic study of chicken populations (Al-Jumaili and Hanotte, 2022). Mitochondrial phylogenies defined by the D-loop sequences have been regarded as sufficiently supported by the complete genomic sequences (Miao et al., 2013). The mtDNA D-loop marker has been used to study the origin, genetic relationship, and population structure of African chickens (Mobegi, 2006), East African chickens (Mwacharo et al., 2011), and chickens in countries including Ghana (Kayang et al., 2015), South Africa (Mtileni et al., 2011; Nxumalo et al., 2020), Madagascar (Herrera et al., 2017), Egypt (Eltanany and Hemeda, 2016), Nigeria (Adebambo, 2009; Lasagna et al., 2020), and Liberia (Tor et al., 2021). These genetic diversity studies are based on the premise that chicken populations showing higher degrees of diversity in the mtDNA D-loop sequences are more distantly related and of different ancestry compared to closely related populations that share a recent common ancestry. It is therefore imperative to evaluate, under this hypothesis, the present extent of genetic diversity in the Ugandan chicken population and the differentiation among them, with a wider geographic scope in the existing traditional production management. This will serve as an essential component to inform improvement in breeding and conservation strategies for future sustainability. Importantly, this study represents the first national insight into the mtDNA diversity of the indigenous chicken population in Uganda, covering a wider landscape.

## Materials and methods

### Sample collection and laboratory procedures

**FIGURE 1:**
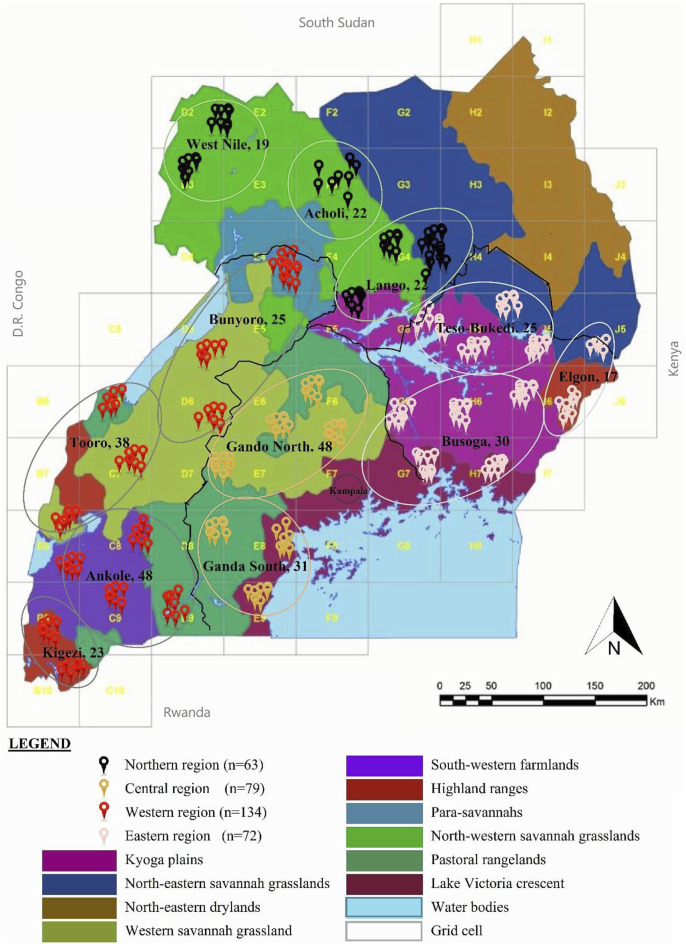
Map of the geographic locations of the indigenous chicken DNA samples in Uganda. The region of DNA samples is represented in a circle. Sample distribution in the randomly selected village households at least 5 km apart, were obtained from grid cells of approximately 50 km^2^ across Uganda to ensure landscape data. Adapted from Figure 1 in Yussif et al. (2023), licensed CC-BY-4.0.

Whole blood samples were drawn from the brachial/ulnar wing vein of 344 indigenous chickens selected from 12 subregion-base chicken populations in 35 districts across the four regions in Uganda (Figure 1). Two mature chickens in each indigenous chicken-keeping household kept under traditional husbandry management were randomly sampled from villages that were at least 5 km apart map: http://tinyurl.com/2s4k4p46. In this way, the tendencies of sampling genetically related individuals were minimized since the chickens were traditionally managed under free-range scavenge feeding conditions that rarely had flock pedigree records.

Owen’s (2011) procedure for genetic-based tests was followed, with two drops (approximately 100 μL) of collected blood added to labeled microtubes. The samples were then stored at −20°C until the DNA was extracted, in accordance with FAO (2011) recommendations.

### DNA extraction, PCR amplification, purification, sequencing, and sequence alignment

Genomic DNA was isolated from whole blood and stored in ethanol (100%) or EDTA, following the Bench Protocol (spin column protocol) designed for nucleated erythrocytes (QIAGEN, 2020). Visual estimation of the genomic DNA profile was done using a UV transilluminator documentation system (G-BOX, Syngene).

The fragment, 800 base pairs (bp) of the mtDNA D-loop region, was amplified using previously reported primer pairs: AV1F2: 5'-AGG​ACT​ACG​GCT​TGA​AAA​GC-3' / CR1b: 5'-CCA​TAC​ACG​CAA​ACC​GTC​TC-3'. PCR amplifications were performed in 25 µL reaction volumes of mix containing 30 ng (in 2 µL) genomic DNA, 0.5 (10 pMol/µL) of each primer pair, and 5 µL of the 5× FIREPol^®^ Master Mix Ready to Load (Solis BioDyne) (Solis BioDyne, 2020). The final reaction volume was adjusted with ultrapure PCR-grade water up to the 25 µL reaction volume. The PCR amplification processes were performed on an Applied Biosystems^®^ Veriti^®^ 96-Well Thermal Cycler (Thermo Fisher Scientific), with the following thermal cycling conditions: initial denaturation at 95°C (1 min), denaturation at 95°C (15 s), annealing at 58°C (1 min), elongation at 72°C (1 min), 35-cycle final extension at 72°C (10 min), and held at 4°C until removed. The PCR products were verified via agarose gel electrophoresis using the following parameters: a 1.5% agarose gel stained with 30 µL of ethidium bromide (EtBr) at 150 V for 40 min detected under UV light. The amplified DNA fragment size was estimated through size comparison with a 1-kb DNA ladder ready-to-load (Solis BioDyne) loaded alongside the PCR products. Purification of the PCR products was done using the PureLink^®^ PCR Purification Kit (Thermo Fisher Scientific Inc.). For the sequencing reactions, 30 µL of each of the purified PCR products and 10 pmol/μL of each of the primers were sent for two-way Sanger sequencing on the *Standard-Seq* platform of Macrogen Europe B.V. (https://dna.macrogen-europe.com).

### Sequence quality control and analyses

Sequence fragments of the chicken mtDNA D-loop region were generated using the two forward and reverse primers, such that the target sequence is effectively read twice as a mechanism for chromatogram quality control. The distal (5′ and 3’) ends of Contig sequences were manually inspected using FinchTV v1.4.0 software (Geospiza, Inc.) to improve the reliability of the sequences formed after pairwise alignments of reads. Aligned sequences with a minimum nucleotide quality of at least 80% were assembled into a consensus sequence using BioEdit v7.2.5 (Hall, 1999). Multiple sequence alignment (MSA) to the mtDNA reference sequence of *Gallus gallus domesticus* (GenBank accession number AP003580) was completed using the MUSCLE algorithm in MEGA version 11 (Tamura et al., 2021). Alignments were refined manually to set nucleotide base pairs at 658 bp (651 bp, excluding sites with gaps), covering the available longest sequence of the mtDNA control region common to all samples to capture all probable polymorphisms and avoid ambiguities in downstream analyses.

### Population genetic diversity analyses

Mitochondrial DNA sequence polymorphism parameters, including the positions and number of polymorphic (segregating) sites (*S*) and the distribution of haplotypes, were measured using DnaSP v6.12.03 (Rozas et al., 2017). This analysis involved nucleotide sequences from the 344 individual indigenous chickens grouped according to their study population/taxa (Figure 1; Supplementary Table S1) with one outgroup (GenBank accession number NC_007235). Within-population diversity parameters; haplotype diversity (*Hd*), with corresponding nucleotide diversity (*π*); and the average number of nucleotide differences between haplotypes (*k*) were estimated using Arlequin version 3.5.2.2 (Excoffier et al., 2005).

### Analysis of molecular variance and genetic differentiation

Intrapopulation and interpopulation genetic differentiations were assessed using analysis of molecular variance (AMOVA) to partition the total genetic variance into components using Arlequin 3.5.2.2 software with 1,000 replications (Excoffier et al., 2005). AMOVA was performed at both regional and population/taxa levels using all population group structures in a region, with each region as an individual group. For within-population analyses, the grouping of the populations was as described in the sampling section: the northern region had three sub-regional populations (Acholi, *n* = 22; Lango, *n* = 22; and West Nile, *n* = 19), of which the central region was tested at two population subgroups (Ganda North, *n* = 48; Ganda South, *n* = 31); the western region was composed of four population subgroups (Bunyoro, *n* = 25; Tooro, *n =* 38; Ankole, *n* = 48; and Kigezi, *n =* 23); and the eastern region had three population subgroups (Busoga, *n* = 30; Teso–Bukedi, *n* = 25; and Elgon, *n* = 17).

Genetic differentiations among the 12 populations were measured by the pairwise F_ST_ value, and the statistical tests of the pairwise F_ST_ values were estimated by permutation analysis using 1,000 replications in Arlequin 3.5.2.2 software (Excoffier et al., 2005). The population comparisons were computed based on the matrix of pairwise F_ST_ data from Arlequin output parsed in R software using the parsing script available at https://nicolawongwaiyee.wordpress.com/2017/01/24/extract-fst-mat-data-from-arlequin-xml-output/.

### Network and phylogenetic analyses

The median-joining (MJ) network analysis (Bandelt et al., 1999) of the haplotypes observed was built using PopART 1.7 (http://popart.otago.ac.nz) to infer the possible relationships among the haplotypes of Ugandan chickens. An insight into the haplogroup classification of the populations and their origin was attained using the sequences representing each previously described haplogroup classification by Miao et al. (2013). The GenBank accession numbers of the chicken mtDNA reference sequences in NCBI (https://blast.ncbi.nlm.nih.gov/Blast.cgi) used in this study include haplogroup-A (GU261700), haplogroup-B (GU261699), haplogroup-C (AB114070), haplogroup-D (GU902203), haplogroup-E (AB114076), haplogroup-E1 (AP003317, GU261686, GU261694, GU261709, GU261713, and HQ857210), haplogroup-E2 (HQ857209), haplogroup-E3 (GU261708, HQ857211, and HQ857212), haplogroup-F (DQ648776), haplogroup-G (GU261719), haplogroup-H (GU261715), haplogroup-I (GU261698), haplogroup-W (GU261706), haplogroup-Y (GU261693), and haplogroup-Z (GU261674).

### Population demographic history

Evidence of population demographic profiles, including past spatial range expansion or a stationary population history, was evaluated through Tajima’s (Tajima, 1989) and Fu’s (Fu, 1997) tests of selective neutrality in DnaSP v6.12.03 (Rozas et al., 2017). Mismatch distribution analysis with 1,000 simulations was used to test the validity of the estimated demographic infinite-site model in Arlequin version 3.5.2.2 (Excoffier et al., 2005) software. The mean absolute error (MAE) was calculated between the observed and expected mismatch distributions to provide support for demographic expansion. Tests of goodness-of-fit, Harpending’s raggedness index, and the test statistic sum of squared differences (SSDs) between the observed and the estimated mismatch distribution with *p*-values were generated under the model of selective neutrality. Mismatch frequency graphs were generated in DnaSP v6.12.03 (Rozas et al., 2017) to provide support for demographic expansion.

## Results

### Genetic diversity and haplotype distribution of mtDNA D-loop sequences of Ugandan chickens

**FIGURE 2:**
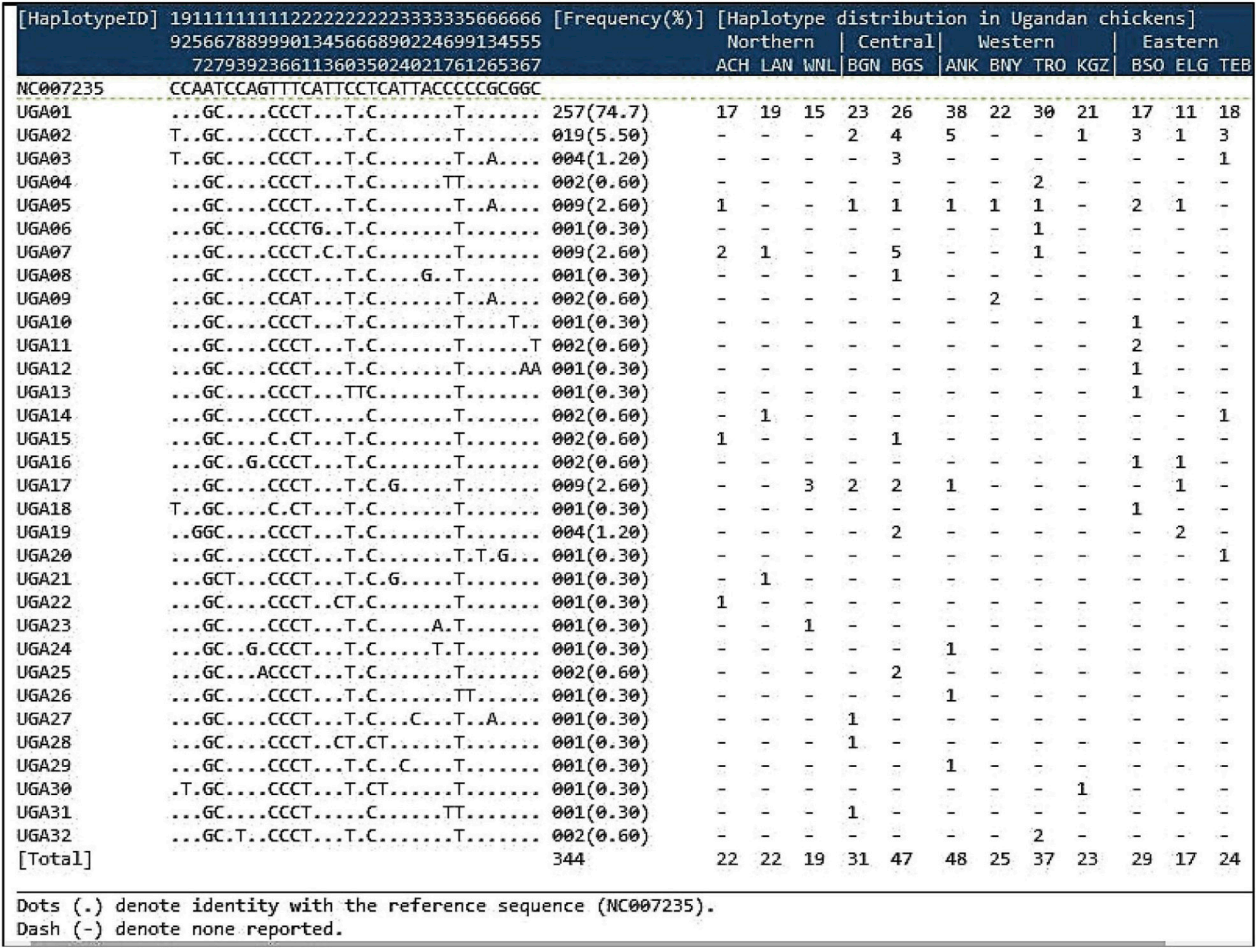
Sequence polymorphism of the 32 haplotypes observed in the D-loop region of 344 Ugandan chickens, along with their frequencies and distribution. Mutations are scored relative to the reference sequence (GenBank accession No. NC007235). Vertically oriented numbers indicate the site, and the sequences shown are only the variable sites.

The results of sequence polymorphism observed in the D-loop region of the 344 isolates and their distribution in the 12 Ugandan chicken populations are presented in Figure 2. The sequences of the individual chicken isolates contained seven alignment gaps, 13 singleton sites (non-informative sites: 92, 179, 231, 263, 304, 320, 322, 341, 592, 613, 646, 654, and 657 bp), and 15 parsimony-informative sites (19, 157, 183, 189, 192, 196, 206, 243, 256, 260, 280, 292, 367, 637, and 658 bp). All the chicken populations showed sequence polymorphisms, with a total of 30 substitutions (4.59%) of 651 nucleotide positions consisting of five transversions and 25 transitions. Twenty-eight (28) variable polymorphic sites (4.26% of the total analyzed loci) defined 32 haplotypes (*H*) named sequentially from UGA01 to UGA32 (Figure 2). The number of haplotypes per population ranged from 3 to 10, with the highest number of haplotypes observed in the Ganda North (n = 10) population (Table 1). A higher (69%) proportion of singleton haplotypes characterized by a single haplotype in a particular population was observed. Haplotype UGA01 was the most frequently observed (74.7%) in all the 12 studied populations. UGA02 occurred at a low frequency (5.5%) in seven populations but was not observed in any of the populations in the northern region. UGA05 was observed (2.6%) in eight populations as singletons. The rest of the haplotypes occurred at very low frequencies, 22 of which were specific to particular regions and populations, while others were shared between populations within and between regions. All observed haplotype (nucleotide) sequences have been deposited at the GenBank with accession numbers OR401589–OR401932 (Supplementary Table S2).

**TABLE 1:** Nucleotide polymorphism of mtDNA D-loop sequences within the indigenous chicken populations in Uganda.

Population	n	V	Pi	S	H	Haplotype diversity *Hd* (SD)	Nucleotide diversity *π* (SD)	K
*Northern*								
Acholi (ACH)	22	4	1	3	5	0.407 (0.128)	0.00068 (0.00024)	0.446
Lango (LAN)	22	4	0	4	4	0.260 (0.120)	0.00056 (0.00029)	0.364
West Nile (WNL)	19	2	1	1	3	0.368 (0.125)	0.00059 (0.00022)	0.386
All	63	8	2	6	9	0.344 (0.077)	0.00062 (0.00016)	0.403
*Central*								
Ganda South (BGS)	31	8	3	5	7	0.452 (0.110)	0.00107 (0.00033)	0.697
Ganda North (BGN)	47	8	6	2	10	0.679 (0.072)	0.00146 (0.00022)	0.947
All	78	13	6	7	13	0.596 (0.064)	0.00131 (0.00019)	0.851
*Western*								
Ankole (ANK)	48	7	1	6	7	0.368 (0.086)	0.00068 (0.00019)	0.441
Bunyoro (BNY)	25	2	2	0	3	0.227 (0.106)	0.00057 (0.00028)	0.373
Tooro (TRO)	37	5	2	3	6	0.344 (0.099)	0.00057 (0.00018)	0.372
Kigezi (KGZ)	23	3	0	3	3	0.170 (0.102)	0.00040 (0.00026)	0.261
All	133	14	5	9	13	0.302 (0.052)	0.00059 (0.00012)	0.385
*Eastern*								
Busoga (BSO)	29	8	3	5	9	0.653 (0.097)	0.00142 (0.00030)	0.921
Elgon (ELG)	17	5	1	4	6	0.588 (0.135)	0.00106 (0.00030)	0.691
Teso–Bukedi (TEB)	24	5	1	4	5	0.435 (0.119)	0.00096 (0.00032)	0.623
All	70	13	5	8	14	0.560 (0.070)	0.00117 (0.00019)	0.762
Overall	344	28	15	13	32	0.437 (0.034)	0.00088 (0.00008)	0.576

n, number of samples; V, variable (polymorphic) sites; Pi, parsimony-informative site; S, singleton site; H, number of haplotypes; SD, standard deviation; k, average number of nucleotide differences.

Generally, the haplotype diversity (*Hd*) was 0.437 ± 0.034, while the nucleotide diversity was 0.00088 ± 0.00008, with the average number of nucleotide differences (k) between haplotypes at 0.576 (Table 1). Both haplotype and nucleotide diversities were highest for the Ganda North population, whereas the Kigezi population showed the lowest genetic diversity. Regionally, the population in the central region altogether showed the highest haplotype (gene) and nucleotide diversities, while the lowest was recorded in the Western chicken population.

### Genetic structure of the indigenous chicken population in Uganda

**FIGURE 3:**
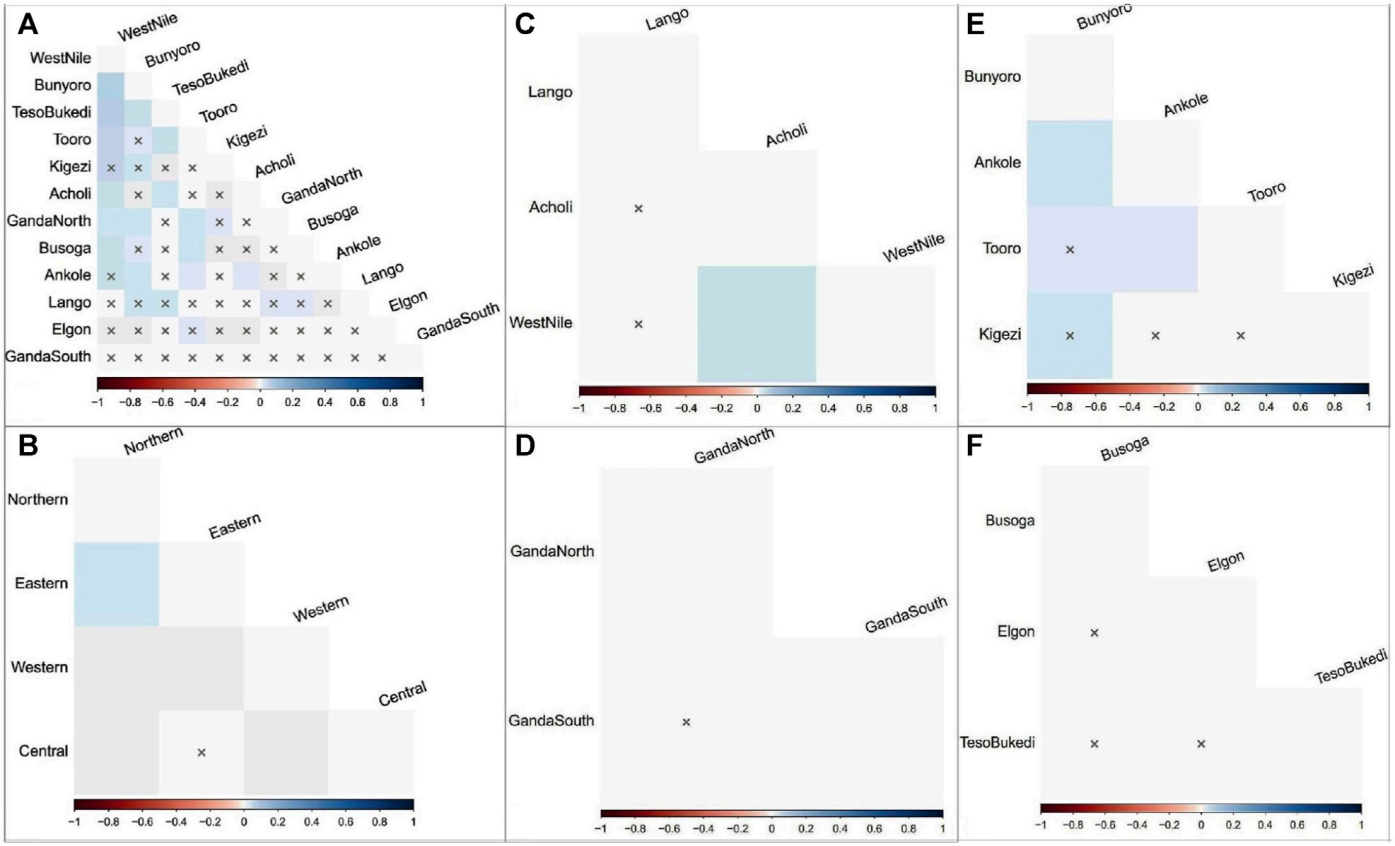
Pairwise F_ST_ matrix representations for population comparison in Ugandan indigenous chickens. Shading Hue reflects the F_ST_ value and degree of divergence among populations at a 0.05% significant level. **(A)** Sub-regions. **(B)** Regions. **(C)** Northern region. **(D)** Central region. **(E)** Western region. **(F)** Eastern region. x, insignificant *p*-value.

The results of AMOVA to assess the genetic structure of the indigenous chicken populations in Uganda are presented in Table 2. The genetic variation observed among chicken individuals within the population was 98.18% (*ρ* < 0.01) of the overall genetic variations, while the percentage of variation among regional groups constituted 1.08% (*ρ* < 0.05), and only 0.75% (*ρ* > 0.05) of the variation accounted for populations within regions. Regionally, the highest percentage of variation was found among chicken individuals within populations for the northern (98.41%), central (99.25%), western (97.81%), and eastern (100.41%) regions. Only the chicken populations in the western region were differentiated (*ρ* < 0.05), with a percentage variation of 2.19% compared to the observed variation among populations in the northern (1.59%), central (0.75%), and eastern (−0.41%) regions. The results are compatible with the matrix of pairwise F_ST_ estimates for all population comparisons in Figure 3 and Supplementary Table S3. The genetic distances between the populations within the central and eastern regions were not significant (ρ > 0.05). Only Acholi and West Nile chicken populations showed a higher genetic distance (ρ < 0.05) in the northern region. However, Ankole and Bunyoro, as well as Ankole and Tooro chicken populations in the western region, were significantly distant (ρ < 0.05).

**TABLE 2:** Analysis of molecular variance based on the frequencies of the 32 haplotypes observed in the Ugandan chicken population.

Source of variation/grouping	d.f.	Variance component	Percentage of variation	Fixation index, F	ρ-value
*Overall*					
Among regions	3	0.00312 Va	1.08	0.01079^CT^	0.03421
Among populations within regions	8	0.00215 Vb	0.75	0.00753^SC^	0.07038
Within population	332	0.28377 Vc	98.18	0.01824^ST^	0.00293
Total	343	0.2894			
Northern					
Among population	2	0.00322 Va	1.59	0.0159^ST^	0.13392
Within population	60	0.19956 Vb	98.41		
Total	62	0.20279			
*Central*					
Among population	1	0.00319 Va	0.75	0.00745^ST^	0.23167
Within populations	76	0.42420 Vb	99.25		
Total	77	0.42738			
*Western*					
Among population	3	0.00423 Va	2.19	0.02189^ST^	0.02346
Within population	129	0.18920 Vb	97.81		
Total	132	0.19343			
*Eastern*					
Among population	2	−0.00155 Va	−0.41	−0.00408^ST^	0.56109
Within population	67	0.38198 Vb	100.41		
Total	69	0.38043			

F_CT_, variation among groups (regions) divided by total variation; F_SC_, variation among populations (sub-regions) divided by the sum of variation among populations within groups and variation within populations; F_ST_, the sum of variation groups divided by total variation; d. f., degrees of freedom.

### Demographic history inferred from mtDNA D-loop sequences of the indigenous chicken population in Uganda

**FIGURE 4:**
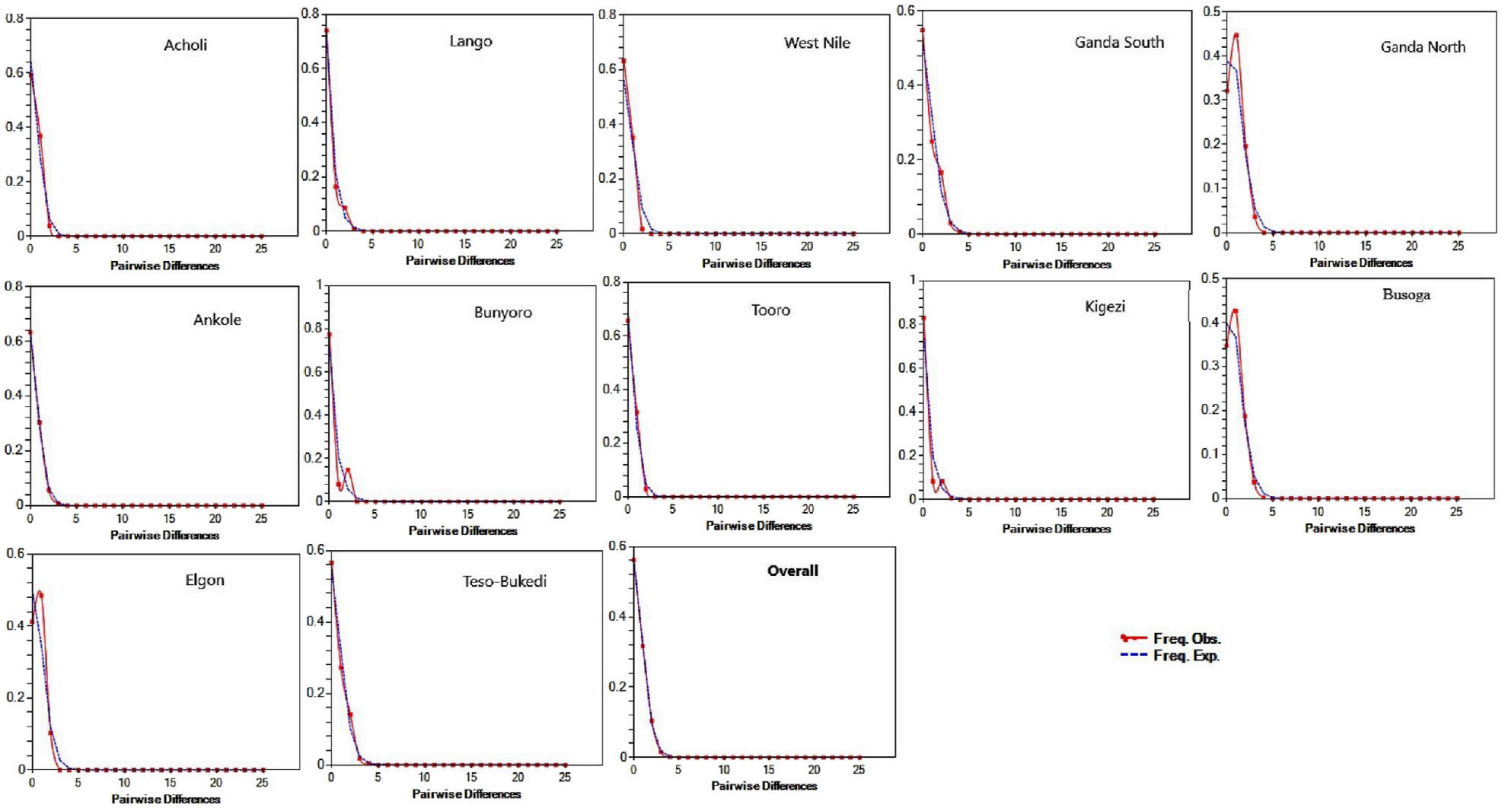
Mismatch distribution patterns for indigenous chicken populations across the sub-regions. The *x*-axis shows the number of pairwise differences, and the *y*-axis shows the frequency of the pairwise comparisons. The observed frequencies were represented by a continuous red line, while the frequency expected under the hypothesis of the population expansion model was depicted by the blue dotted line.

Historical demographic patterns of genetic variation in the indigenous chicken population based on neutrality tests augmented with mismatch distribution are presented in Table 3 and Figure 4. The MAE values were moderate to low. Overall, Tajima’s D and Fu’s Fs values obtained were negative, indicating an excess (*ρ* < 0.05) of rare mutations in the chicken population and providing evidence of population expansion. All the chicken populations deviated (*ρ* < 0.05) from neutrality except for the West Nile and Bunyoro populations, which did not clearly support deviation (*ρ* > 0.05) from neutrality. Therefore, the hypothesis of neutral evolution was significantly rejected for all populations, except for West Nile and Bunyoro populations due to the insignificant Tajima’s D and Fu’s Fs values. The sum of squared differences (SSDs) and Harpending’s raggedness indices (r) were not significant at the population group level, indicating that the mismatch distribution curves fit the sudden expansion model tested. This is consistent with the unimodal patterns of the mismatch distribution graphs observed in all the studied populations (Figure 4) and the results of sudden demographic expansion in Table 3.

**TABLE 3:** Neutrality and demographic expansion indices.

Population	MAE	Tajima’s D (ρ)	Fu’s Fs (ρ)	SSD (ρ)	Harpending’s r (ρ)
*Northern*					
Acholi (ACH)	0.3094	−1.667 (0.028)	−3.200 (0.002)	0.00512 (0.00001)	0.1604 (1.000)
Lango (LAN)	0.0827	−1.878 (0.007)	−2.205 (0.003)	0.00045 (0.37600)	0.3437 (0.872)
West Nile (WNL)	0.2999	−0.778 (0.234)	−0.725 (0.196)	0.00597 (0.01000)	0.1902 (0.010)
All	0.1679	−1.994 (0.001)	−8.877 (<0.001)	0.00021 (0.24900)	0.1935 (0.621)
*Central*					
Ganda South (BGS)	0.1473	−1.966 (0.006)	−4.077 (0.004)	0.00127 (0.69300)	0.1154 (0.8080)
Ganda North (BGN)	0.5432	−1.317 (0.101)	−5.893 (0.001)	0.00922 (0.12000)	0.1056 (0.0340)
All	0.3863	−1.892 (0.007)	−9.808 (<0.001)	0.00093 (0.14300)	0.0647 (0.5370)
*Western*					
Ankole (ANK)	0.1818	−1.922 (0.002)	−5.310 (0.000)	0.00027 (0.24900)	0.1714 (0.6680)
Bunyoro (BNY)	0.2359	−0.640 (0.272)	−0.607 (0.227)	0.00504 (0.25400)	0.5067 (0.8320)
Tooro (TRO)	0.2352	−1.785 (0.011)	−4.221 (0.001)	0.00001 (0.91300)	0.1992 (0.8210)
Kigezi (KGZ)	0.1720	−1.731 (0.021)	−1.305 (0.034)	0.00120 (0.30600)	0.5643 (0.8730)
All	0.0754	−2.240 (<0.001)	−15.732 (<0.001)	0.00011 (0.51500)	0.2565 (0.8270)
*Eastern*					
Busoga (BSO)	0.4881	−1.677 (0.030)	−5.508 (<0.001)	0.00134 (0.63000)	0.0584 (0.3510)
Elgon (ELG)	0.4873	−1.718 (0.020)	−3.771 (<0.001)	0.01777 (0.00001)	0.1622 (0.0400)
Teso–Bukedi (TEB)	0.1714	−1.549 (0.042)	−2.139 (0.024)	0.00124 (0.83000)	0.1185 (0.6400)
All	0.3627	−2.038 (0.004)	−13.043 (<0.001)	0.00023 (0.48400)	0.0666 (0.5710)
Overall	0.2081	−2.319 (<0.001)	−51.369 (<0.001)	0.00016 (0.56900)	0.1138 (0.7580)

n, number of samples; S, variable (polymorphic) sites; SD, standard deviation; MAE, mean absolute error; Tajima’s D and Fu’s Fs are tests of selective neutrality; SSD, sum of squared deviations; r, raggedness index; ρ, ρ-value.

### Haplotype network and phylogenetic relationships among the Ugandan chicken population

**FIGURE 5:**
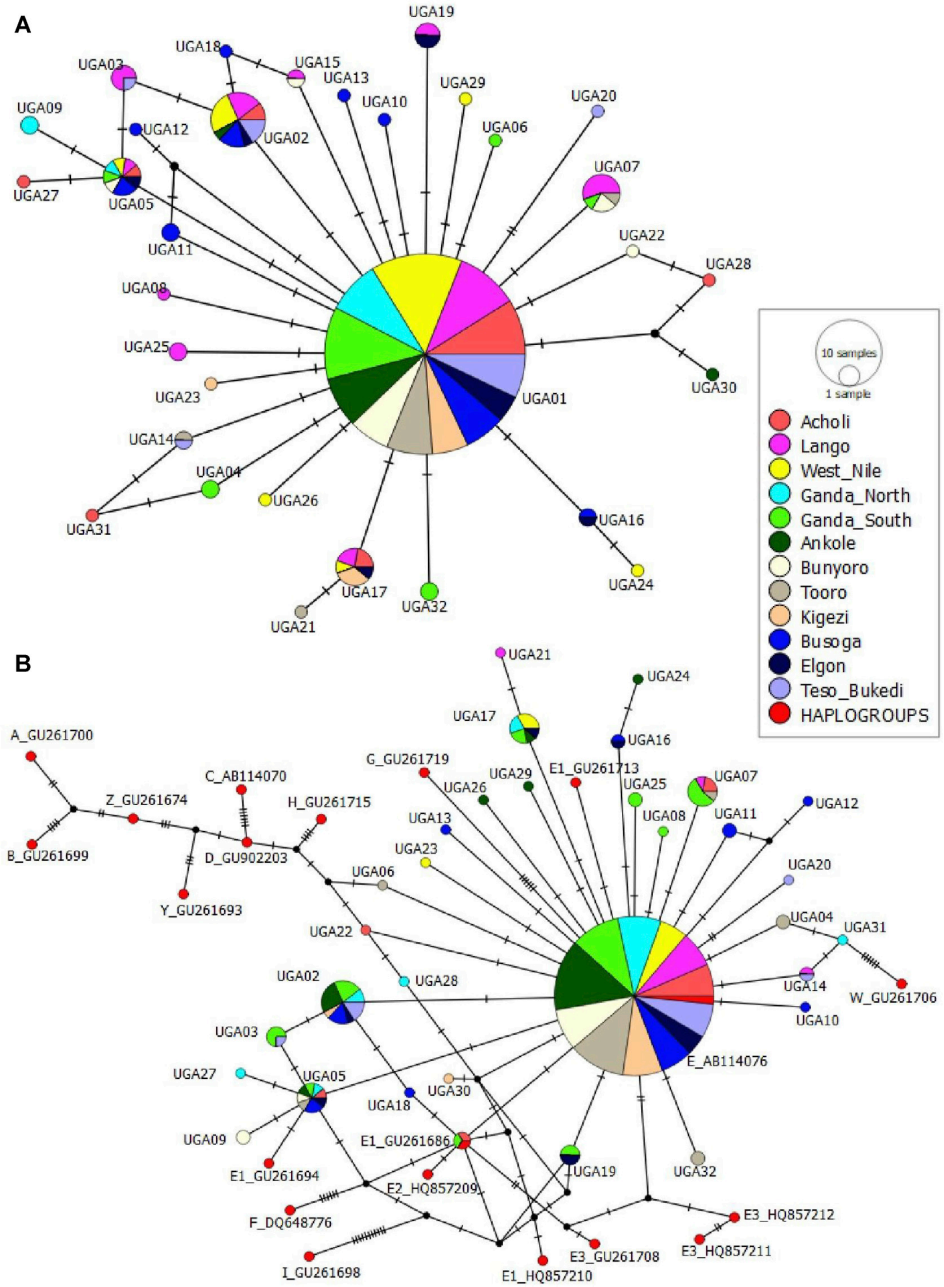
**(A)** Median-joining [MJ] network profile of the 32 mtDNA D-loop haplotypes observed in 344 Ugandan chicken population **(B)** MJ network showing the relationship between the 32 haplotypes and 15 chicken mtDNA reference haplogroup classification. Based on data from Miao et al. (2013) from the GenBank. The area of the circles is proportional to haplotype frequency and the hatch marks on the line correspond to mutational positions connecting haplotypes. Median vectors are represented by black circles.

**FIGURE 6:**
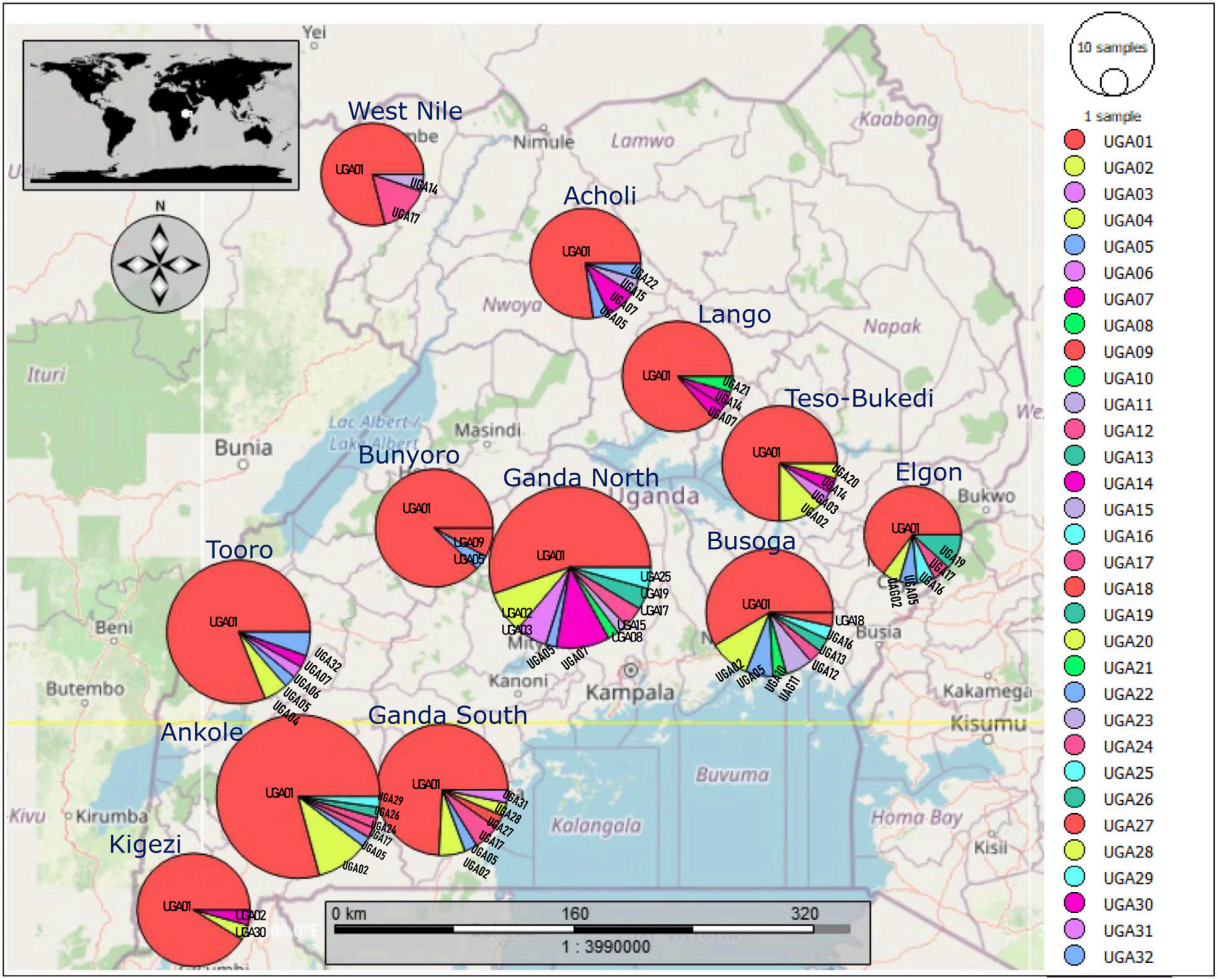
Geographic distribution of the 32 haplotypes observed in the mtDNA D-loop sequences of the indigenous chickens in Uganda.

The median-joining network profile of the mtDNA D-loop sequences observed in the Ugandan indigenous chicken populations revealed that all the haplotypes radiate from the haplotype UGA01 sequence (Figure 5A) and showed affinity to the haplogroup E1 from lineage E (Figure 5B) of the reference haplogroup classification. The divergence between the observed haplotypes and all the other reference haplogroups was well-resolved. Most of the haplotypes were separated from UGA01 by a single mutation, with a few being separated by two or three mutations. However, the link between UGA12 and UGA30 was not well-resolved. UGA12 and UGA30 were connected to UGA01 by one median vector each, which could be either haplotypes not sampled, never introduced into Uganda, or at the brink of extinction. UGA01 had the largest geographic distribution, shared across Uganda (Figure 6), indicating a close affinity of all haplotypes to haplogroup E1 of Miao et al. (2013) nomenclature.

## Discussion

Investigating the underlying molecular genetic diversity within the Ugandan indigenous chicken population, which has had a close link to its heritage for numerous years, is essential.

This could unravel information pertinent to improving selection designs, breeds, and conservation efforts adapted to local conditions (AU-IBAR, 2019; Hvilsom et al., 2022). An mtDNA D-loop has been used as a marker to infer molecular diversity and population differentiation in various species due to minimal recombination (Lan et al., 2015); as such, it can be clonally inherited, with neutral or near-neutral molecular evolution. Hence, any polymorphism that arises in the D-loop can be precisely situated in the phylogenetic tree in the form of haplotypes and their corresponding haplogroups. This study provides the first national approach to present mtDNA D-loop sequence analyses to evaluate the genetic diversity of the Ugandan chicken population and their phylogenetic and phylogeographic structures.

As shown, analysis of the mtDNA D-loop sequences detected the site of genetic polymorphism between 19 and 567 bp, which is consistent with the region typically reported in most studies (Liu et al., 2006; Al-Jumaili et al., 2020; Lasagna et al., 2020). Estimates of haplotype (gene) and nucleotide diversities correlate with the degree of genetic variation in the population and are, hence, indicative of genetic diversity (Frankham et al., 2010). The haplotype and nucleotide diversity estimates of the chicken populations detected were low but similar to earlier reports for Teso, Langi, Nganda, and Nkonjo chicken populations in Uganda, as well as in Ethiopian and Sudanese populations (Mwacharo et al., 2011). Similarly, low haplotype diversity was observed in Egyptian (Eltanany and Hemeda, 2016), Nigerian (Adebambo et al., 2010; Lasagna et al., 2020), and Zimbabwean (Muchadeyi et al., 2008) chicken populations. Within Uganda, the chicken populations from the central and eastern regions showed more genetic variation than the northern and western populations, as reflected in the lower polymorphism in the mtDNA control region of the West Nile, Bunyoro, and Kigezi populations (Table 1). This may be related to a higher degree of inbreeding within the northern and western populations. Hence, the chicken populations in both the central and eastern regions have relatively richer gene pools and higher selection potential to be explored for sustainable utilization and conservation.

The analysis of sequence variations AMOVA revealed genetic differentiation only among chicken haplotypes (individuals) within populations. The premium for ease of adaptability to the production environment, the traditional value of the chickens, and the quality of products incentivize indigenous chicken production for farmers, highlighting the importance of within-population diversity. The Western region populations showing genetic differences (ρ < 0.05) corroborate the results of the pairwise F_ST_ estimates in Figure 3 and Supplementary Table S3. Higher (*ρ* < 0.05) genetic distances were observed between Ankole and Bunyoro and in Ankole and Tooro compared to the rest of the Western Ugandan populations, which showed no significant (*ρ* > 0.05) paired distances. Population fragmentation, likely as a result of the restricted gene flow, inbreeding, genetic drift, and small population size (Frankham et al., 2010), in the Western region might be the probable cause signaling the population differences in the region. In addition, the degree of genetic differentiation among populations is expected to be greater for subdivided populations, which was not the case in this study. Furthermore, the chicken population in the western region showed the lowest genetic diversity estimates (Table 1) in this study. An earlier phenotypic study in Uganda found the chicken population in the Western region to be lighter in weight with a higher rate of inbreeding compared to the other three regions (Yussif et al., 2023). The non-significant negative F_ST_ estimate obtained among the population in the eastern region is indicative of an absence of the population genetic structure. The relatively low genetic distances (pairwise F_ST_ estimates) observed among the population, with only a few being genetically distant (*ρ* < 0.05), demonstrate genetic intermixing among the chicken populations within the country.

The signal of genetic differentiation in the West Nile and Bunyoro chicken populations is consistent with population variation in neutral mutation, indicating likely evidence of a population that has undergone a bottleneck effect. Neutral mutations account for a proportion of genetic diversity whose fate is determined by random genetic drift (Frankham et al., 2010). Moreover, the overall significant negative Tajima’s D and Fu’s Fs estimates of the chicken population provided evidence for deviation from neutrality, as expected in a population undergoing expansion. The unimodal mismatch distribution pattern (Figure 4) and non-significant raggedness index (Table 3) observed in all studied populations further confirm population expansion following arrival. Generally, beneficial genetic variation is accumulated and maintained in a rapidly expanding population (Frankham et al., 2010).

The MJ network analysis of the mtDNA D-loop haplotypes of the Ugandan chicken population revealed a dominant UGA01 haplotype shared by all the 12 geographically defined populations, from which all haplotypes diverged in a “star-like” topology (Figure 5A). This suggests that UGA01 is an ancestral haplotype in Uganda, indicating a close relationship among all the haplotypes. In addition, an association of one common haplotype with others of lower frequencies or private haplotypes is compatible with populations that have undergone expansion after arrival. Furthermore, the high ratio of singleton haplotypes is indicative of a population expansion from a small number of founders. Extensive south–north population dispersal from the Lake Victoria crescent and genetic intermixing, following human movement and ancient commercial activities in the area (Hartwig, 1970), cannot be disregarded in the distribution of the haplotypes. Moreover, the traditional breeding practices where breeding stocks, especially mature hens and pullets, are obtained from neighbors and/or the communal market centers may have contributed to the extensive dispersal of the ancestral haplotype, UGA01. Furthermore, the MJ network of the haplotypes with reference haplotypes (Miao et al., 2013) showed the clustering of the Ugandan chickens in one major haplogroup, E1 of E-lineage (Figure 5B). Haplogroup-E1 is believed to have its center of diversity in the Indian subcontinent and East Asia. It is the most widely distributed lineage globally and the commonest in Africa (Miao et al., 2013), notably reported in Liberia (Tor et al., 2021), Nigeria (Lasagna et al., 2020), Egypt (Eltanany and Hemeda, 2016), Algeria Ethiopia (Mwacharo et al., 2011; Al-Jumaili et al., 2020; Al-Jumaili et al., 2020), Tanzania (Lyimo et al., 2013), and South Africa (Mtileni et al., 2011; Nxumalo et al., 2020).

The mtDNA D-loop phylogenetic relationship observed in this study is consistent with a lack of phylogeographic structure among the different populations sampled across the country. A haplotype network characterized by an expansion from a single ancestral haplotype, as observed in this study, is associated with a single geographic origin. The belief of Egypt, which mainly has the lineage-E haplogroup, as one of the entry points of the haplogroup-E lineage in Africa (Eltanany and Hemeda, 2016), therefore, cannot exclude an ancient single origin for the Ugandan chicken population. Interestingly, an earlier regional study suggested the haplogroup-D lineage of chickens sampled from Uganda (Mwacharo et al., 2011). However, a reanalysis of the mtDNA sequence of Nigerian chickens with other African populations linked all the Ugandan chickens to the haplogroup-E lineage (Lasagna et al., 2020), supporting the claim of this current study. Moreover, all the chicken populations in this current study clustered with both the Ugandan samples (GenBank accessions: EU095034–EU095192) in the research by Mwacharo et al. and the reference haplogroup E classification established by Miao et al. (2013). Hence, the Ugandan chicken population is most probably of the haplogroup-E lineage without any significant phylogeographic substructure in Uganda; this may be a consequence of the high gene flow (Frankham et al., 2010) from the breeding and management practices (Yussif et al., 2023) prevailing across Uganda.

This study, together with a preceding study (Yussif et al., 2023), demonstrates the significance of a harmonized approach to AnGR characterization, where information on a production environment could be combined with phenotypic and molecular genetic data to guide decision-making. Indigenous chicken production under traditional husbandry conditions is characterized by the absence of pedigree data and records of origin. This suggests that molecular marker data are useful in providing the most reliable genetic diversity estimates within a set of populations. This information could be useful in identifying a careful balance between adaptability, farmer preference, and optimal management strategies in the sustainable development of indigenous chickens in Uganda.

## Conclusion

Ugandan chicken populations exhibit significant genetic similarity, with no detectable genetic structuring among them. However, there is a noticeable presence of genetic diversity among the central and eastern chicken populations, with high within-population genetic differentiation. The chicken population is of a single phylogenetic lineage, haplogroup-E, from the Indian subcontinent. Hence, combined with their adaptive presence in Uganda, they represent a genetic resource that should be preserved for the refinement of breeding and conservation strategies, together with policy directions on breeding management practices across the country. For instance, crossbreeding should be guided by selective improvement of the chickens, focused mainly on upgrading production traits with a nominal emphasis on esthetics. The northern and western chicken populations should be genetically improved alongside management strategies to re-establish the historical gene flow among the fragmented population to create a balanced genetic diversity that will improve population fitness and productivity. In addition, strategies should be focused on upgrading this valuable resource with interventions aimed at safeguarding the genetic diversity, particularly of the central and eastern populations, for sustainable utilization.

## Data Availability

The datasets presented in this study can be found in online repositories. The names of the repository/repositories and accession number(s) can be found in the article/Supplementary Material.
