# Supplementary material for: Uganda chicken genetic resources: II. genetic diversity and population demographic history inferred from mitochondrial DNA D-loop sequences
Source: Front Genet. 2024 Mar 7;15:1325569. doi: 10.3389/fgene.2024.1325569 (PMC10955702; doi:10.3389/fgene.2024.1325569)
Supplement: Supplementary file 2 [file Table1.DOCX]

**Table S 1: Study population, location and sample size**

| **Population/Sub-regions** | **Districts (Locations)** | **Sample size (n)** |
| --- | --- | --- |
| ***Northern region*** |  | *63* |
| Acholi (ACH) | Omoro, Amuru | 22 |
| Lango (LAN) | Apac, Lira | 22 |
| West Nile (WNL) | Maracha, Yumbe | 19 |
|  |  |  |
| ***Central region*** |  | *78* |
| Ganda North (BGN) | Mubende, Nakaseke, Nakasongola, Luwero | 47 |
| Ganda South (BGS) | Rakai/Kyotera, Masaka, Ssembabule | 31 |
| ***Western region*** |  | *133* |
| Ankole (ANK) | Mbarara, Kiruhura, Buhweju, and Isingiro | 48 |
| Bunyoro (BNY) | Hoima, Kiryandongo, Kibale | 25 |
| Tooro (TRO) | Kabarole, Ntoroko, Kasese | 37 |
| Kigezi (KGZ) | Kabale, Rukungiri, | 23 |
| ***Eastern region*** |  | *70* |
| Busoga (BSO) | Kamuli, Iganga, Jinja, Bugiri | 29 |
| Elgon (ELG) | Kapchorwa, Mbale | 17 |
| Teso/Bukedi (TES) | Serere, Amuria, Kumi, Budaka | 24 |
| **Total** | **35** | **344** |
| Four (4) regions, sub-grouped into twelve populations/ taxa | | |
