# Supplementary material for: Uganda chicken genetic resources: II. genetic diversity and population demographic history inferred from mitochondrial DNA D-loop sequences
Source: Front Genet. 2024 Mar 7;15:1325569. doi: 10.3389/fgene.2024.1325569 (PMC10955702; doi:10.3389/fgene.2024.1325569)
Supplement: Supplementary file 3 [file Table3.DOCX]

[**Table S3:**](#_5.3.2_Genetic_structure) **Population pairwise F_ST_ values (Distance method: Pairwise difference)**

| **Chicken populations** | **F_ST_ values *ρ*-values** | | | |
| --- | --- | --- | --- | --- |
| *Region* |  |  |  |  |
|  | Northern | Central | Western | Eastern |
| Northern |  | 0.02734 | 0.01758 | 0.00000 |
| Central | **0.01603**^*^ |  | 0.00781 | 0.41309 |
| Western | **0.01237**^*^ | **0.01574**^*^ |  | 0.01758 |
| Eastern | **0.03082**^*^ | -0.00021 | 0.01339 |  |
| The lower diagonal shows pairwise F_ST_ estimates  Upper diagonal presents (ρ-values) of pairwise F_ST_ values.  ^*^*ρ<*0.05 | | | | |

**Table S3 (continue): Population pairwise FSTs difference**

| **Chicken populations** | **F_ST_ values *ρ*-values** | | | |
| --- | --- | --- | --- | --- |
| *Northern region* |  |  |  |  |
|  | Acholi | Lango | West Nile |  |
| Acholi |  | 0.9990 | 0.04004 |  |
| Lango | -0.00982 |  | 0.30957 |  |
| West Nile | **0.04922**^*^ | 0.00867 |  |  |
| *Central region.* | | | | |
|  | Ganda North | Ganda South |  |  |
| Ganda North |  | 0.2548 |  |  |
| Ganda South | 0.00745 |  |  |  |
| *Western region* | | | | |
|  | Ankole | Bunyoro | Tooro | Kigezi |
| Ankole |  | 0.03711 | 0.02344 | 0.53418 |
| Bunyoro | **0.03809**^*^ |  | 0.1113 | 0.11719 |
| Tooro | **0.02498**^*^ | 0.02576 |  | 0.27637 |
| Kigezi | -0.00592 | 0.03948 | 0.00623 |  |
| *Eastern region* | | | | |
|  | Busoga | Elgon | Teso-Bukedi |  |
| Busoga |  | 0.43359 | 0.67969 |  |
| Elgon | -0.00208 |  | 0.27051 |  |
| Teso-Bukedi | -0.01145 | 0.00789 |  |  |
| The lower diagonal shows pairwise F_ST_ estimates  The upper diagonal presents ρ-values of pairwise F_ST_ values.  ^*^*ρ<*0.05 | | | | |

**Table S3 (continue): Population pairwise FSTs (Distance method: Pairwise difference)**

| **Chicken populations** | **F_ST_ values *ρ*-values** | | | | | | | | | | | |
| --- | --- | --- | --- | --- | --- | --- | --- | --- | --- | --- | --- | --- |
|  | BGN | BGS | ANK | BNY | TRO | KGZ | ACH | LAN | WNL | BSO | ELG | TEB |
| Ganda North (BGN) |  | 0.22168 | 0.11816 | 0.04199 | 0.00781 | 0.10449 | 0.25586 | 0.11914 | 0.04688 | 0.26660 | 0.49805 | 0.42969 |
| Ganda South (BGS) | 0.00745 |  | 0.53027 | 0.28223 | 0.27246 | 0.73340 | 0.45605 | 0.39453 | 0.72070 | 0.31250 | 0.75684 | 0.46582 |
| Ankole (ANK) | 0.01375 | -0.00375 |  | 0.04297 | 0.01758 | 0.57812 | 0.04883 | 0.15527 | 0.05566 | 0.30176 | 0.43359 | 0.63086 |
| Bunyoro (BNY) | **0.03156**^*^ | 0.00544 | **0.03809**^*^ |  | 0.09863 | 0.13867 | 0.17676 | 0.13477 | 0.02832 | 0.10742 | 0.24121 | 0.03418 |
| Tooro (TRO) | **0.03871**^*^ | 0.00639 | **0.02498**^*^ | 0.02576 |  | 0.27637 | 0.42676 | 0.43555 | 0.01172 | 0.00391 | 0.06641 | 0.01270 |
| Kigezi (KGZ) | 0.02201 | -0.00877 | -0.00592 | 0.03948 | 0.00623 |  | 0.13086 | 0.49805 | 0.06836 | 0.20215 | 0.24805 | 0.34863 |
| Acholi (ACH) | 0.00774 | 0.0005 | **0.02506**^*^ | 0.01679 | 0.00163 | 0.01269 |  | 0.99902 | 0.04297 | 0.10645 | 0.23047 | 0.04785 |
| Lango (LAN) | 0.02088 | -0.00827 | 0.01421 | 0.03482 | 0.00126 | 0.00033 | -0.00982 |  | 0.31641 | 0.05371 | 0.33105 | 0.07812 |
| West Nile (WNL) | **0.03916**^*^ | 0.00067 | 0.04027 | 0.07556 | 0.05212 | 0.05407 | 0.04922 | 0.00867 |  | 0.01855 | 0.21484 | 0.01562 |
| Busoga (BSO) | 0.00499 | 0.0027 | 0.00211 | 0.02539 | **0.03698**^*^ | 0.0112 | 0.01972 | 0.02716 | **0.04655**^*^ |  | 0.44727 | 0.67480 |
| Elgon (ELG) | -0.00351 | -0.01318 | 0.00286 | 0.01889 | 0.02556 | 0.01257 | 0.01432 | 0.00835 | 0.01359 | -0.00208 |  | 0.27539 |
| Teso-Bukedi (TEB) | -0.00161 | -0.00261 | -0.0079 | **0.04854**^*^ | **0.04926**^*^ | 0.01526 | **0.03914**^*^ | 0.03377 | **0.06814**^*^ | -0.01145 | 0.00789 |  |
| The lower diagonal shows pairwise F_ST_ estimates  Upper diagonal presents (ρ-values) of pairwise F_ST_ values.  ^*^*ρ<*0.05 | | | | | | | | | | | | |
